# Methylome changes in *Lolium perenne* associated with long-term colonisation by the endophytic fungus *Epichloë* sp. *Lp*TG-3 strain AR37

**DOI:** 10.3389/fpls.2023.1258100

**Published:** 2023-09-22

**Authors:** Flavia Pilar Forte, Marta Malinowska, Istvan Nagy, Jan Schmid, Paul Dijkwel, David E. Hume, Richard D. Johnson, Wayne R. Simpson, Torben Asp

**Affiliations:** ^1^ Center for Quantitative Genetics and Genomics, Faculty of Technical Sciences, Aarhus University, Aarhus, Denmark; ^2^ Ferguson Street Laboratories, Palmerston North, New Zealand; ^3^ School of Fundamental Sciences, Massey University, Palmerston North, New Zealand; ^4^ AgResearch, Grasslands Research Centre, Palmerston North, New Zealand

**Keywords:** *Lolium perenne*, *Epichloë* sp., DNA methylation, endophytic fungi, drought stress, plant-microbe interactions, artificial association, generation effect

## Abstract

*Epichloë* spp. often form mutualistic interactions with cool-season grasses, such as *Lolium perenne*. However, the molecular mechanisms underlying this interaction remain poorly understood. In this study, we employed reduced representation bisulfite sequencing method (epiGBS) to investigate the impact of the *Epichloë* sp. *Lp*TG-3 strain AR37 on the methylome of *L. perenne* across multiple grass generations and under drought stress conditions. Our results showed that the presence of the endophyte leads to a decrease in DNA methylation across genomic features, with differentially methylated regions primarily located in intergenic regions and CHH contexts. The presence of the endophyte was consistently associated with hypomethylation in plants across generations. This research sheds new light on the molecular mechanisms governing the mutualistic interaction between *Epichloë* sp. *Lp*TG-3 strain AR37 and *L. perenne*. It underscores the role of methylation changes associated with endophyte infection and suggests that the observed global DNA hypomethylation in *L. perenne* may be influenced by factors such as the duration of the endophyte-plant association and the accumulation of genetic and epigenetic changes over time.

## 1 Introduction

*Epichloë* spp. are endophytic fungi that, since the Eocene era, have established a broad spectrum of symbiotic interactions with cool-season grasses, ranging from pathogenic to mutualistic (Schardl and Leuchtmann, 2005; Schardl et al., 2008; Glémin and Bataillon, 2009; Eaton et al., 2015). The type of relationship between endophyte and host plant can vary depending on the fungal reproduction mode (vertical or horizontal transmission) (Schardl and Leuchtmann, 2005) and the genotypes of the symbionts (Christensen et al., 1997; Gagic et al., 2018). When not pathogenic, the fungus is exclusively transmitted to the next generation through seed (Philipson and Christey, 1986). Endophyte-host interactions play a crucial role in plant growth and development and in the response of infected plants to stressors (Schardl and Leuchtmann, 2005).

When in a mutualistic relationship with their host plants, endophytes like *Epichloë* sp. can provide numerous benefits to the host, including protection against biotic and abiotic stressors (Johnson et al., 2013; Hume et al., 2016; Xu et al., 2017). For example, endophytes can produce secondary metabolites that deter herbivores and protect the plant from diseases (Schardl et al., 2008; Bharadwaj et al., 2020). Additionally, endophytes can help plants tolerate abiotic stress, such as drought, by improving water uptake and transport, reducing transpiration, and modulating gene expression (Johnson et al., 2013; Hume et al., 2016; Xu et al., 2017).

The *Lolium perenne-Epichloë* sp. association is a well-studied example of a grass-*Epichloë*- mutualism. While plant and fungal genes that may contribute to the compatibility between partners and the fungal transgenerational transmission in the host have been identified (Gagic et al., 2018), the molecular mechanisms involved in this compatibility are not fully understood. Previous studies have attempted to explore these mechanisms, but further research is needed to gain a deeper understanding (Dupont et al., 2015; Dinkins et al., 2017; Schmid et al., 2017; Bharadwaj et al., 2020). Knowledge about the molecular mechanisms underlying these interactions can provide insights into how endophytes and their host plants interact and can potentially lead to developing strategies for improving plant growth and stress tolerance.

As sessile organisms, plants cannot escape the ever changing and frequently unfavorable environmental conditions they are exposed to. As a result, they evolved to have sophisticated mechanisms of gene regulation to ensure rapid response to stressors that allow them to survive environmental fluctuations. Environmental cues are sensed and translated into cellular responses through changes in signal transduction pathways and altered gene expression, all of which can result in physiological and morphological adaptation to stress conditions (Mirouze and Paszkowski, 2011; Lamers et al., 2020).

Epigenetics refers to modifications that do not involve changes to the underlying DNA sequence but can still affect gene expression. These modifications, including DNA methylation, histone modification, and small RNA interactions, play a role in plant defense responses, the establishment of symbiosis, and other plant-organism interactions (Alonso et al., 2019; Ramos-Cruz et al., 2021). DNA methylation has been linked to defense against pests in plants, and a widespread loss of methylation (hypomethylation) has been shown to enhance resistance to certain pathogens (Akimoto et al., 2007; Stroud et al., 2013; Yu et al., 2013). DNA methylation has also been shown to be important in forming nitrogen-fixing nodules in legumes and the response of soybean to nematode infection (Rambani et al., 2015; Hewezi et al., 2017; Rambani et al., 2020). In beneficial interactions, such as arbuscular mycorrhizal fungi colonization, DNA methylation levels can increase or decrease depending on the specific interaction (Cicatelli et al., 2014; Varga and Soulsbury, 2017; Varga and Soulsbury, 2019). In addition to DNA methylation, histone modifications also play a role in plant defense responses, with acetylation and deacetylation regulating gene expression in response to pests and pathogens (Yuan et al., 2013; Kang et al., 2022). Small RNAs, including miRNAs and siRNAs, have also been linked to plant defense, with their levels and activity altered in response to biotic stress (Ruiz-Ferrer and Voinnet, 2009; Cai et al., 2018; Hou et al., 2019). Epigenetic modifications can also be influenced by environmental factors, including temperature and the availability of nutrients (Karan et al., 2012; Luo et al., 2012; Ferreira et al., 2019; Sun et al., 2022).

Epigenetic mechanisms are involved in both defense and susceptibility in plant-pathogen interactions. However, the precise mechanisms by which epigenetic modifications contribute to these interactions are still being studied (Lippman et al., 2004; Pavet et al., 2006; Dowen et al., 2012; Yu et al., 2013; Zhu et al., 2016). There is also evidence that plant epigenetic makeup can influence its microbiome composition and that plant-microbe associations can impact plant epigenetic modifications (Chen et al., 2022).

This study aimed to investigate the effect of the endophytic fungus *Epichloë* sp. *Lp*TG-3 strain AR37 (Johnson et al., 2013) on DNA methylation of its host plant, *L. perenne*, over multiple generations. While some studies have investigated the role of epigenetic modifications, such as histone methylation, in *Epichloë*-grass interactions, most of the research has focused on the fungus rather than the impact on DNA methylation in the host plant (Chujo and Scott, 2014). Our objective was to examine the changes in DNA methylation associated with *Epichloë* infection in *L. perenne* and elucidate their potential implications for the host plant.

## 2 Methods

### 2.1 Biological material

Biological material for this study included perennial ryegrass seeds (*L. perenne* L. cv Grasslands Samson) obtained from the Margot Forde Germplasm Centre (Palmerston North, New Zealand). The seeds were infected with the endophytic fungus *Epichloë* sp. *Lp*TG-3 strain AR37 as described by (Johnson et al., 2013) and were from the second, sixth and ninth generation of a seed production program designed to maintain high levels of endophyte infection (designated as G2, G6, and G9, respectively). The methods for seed germination and plant growth were previously described by Forte et al. (Forte et al., 2020).

Briefly, 135 to 180 seeds were germinated in propagation trays for each generation. After three weeks, seedlings were transplanted into 1 L pots containing potting mix augmented with dolomite and fertilizer. The plants were grown in the glasshouse under natural daylight. Every six months, plants were clonally propagated to reach the desired number of individual plants, and the endophyte infection status (E- for endophyte-negative or E+ for endophyte-positive) was confirmed in three tillers per plant using a tissue-print immunoassay as previously described (Simpson et al., 2012).

### 2.2 Endophyte status of the plants

The three analyzed generations of *L. perenne* came from an original small population (G0; n=38) artificially infected with AR37 at AgResearch in 1996 (Forte et al., 2020). This means that un-inoculated *L. perenne* plants, serving as true negative controls, were not included in this study. All E- samples used in the experiment come from plants that spontaneously lost the endophyte at some point in the seed propagation program. However, in our experiment, the E- individuals within each generation represent a state without current endophyte infection, allowing for a comparison of the methylation patterns between endophyte-infected (E+) and endophyte-negative (E-) individuals within each generation.

### 2.3 Water deficit experimental setup

Experiments investigating the effect of drought stress on plants were conducted under controlled environmental conditions. Plants were randomly selected from within the populations G2, G6, and G9, with three E+ and three E- plants from G2 and G6, and three E+ and two E- plants from G9. Five tillers from each plant were potted in 300 ml trapezoidal root trainers (Flight-Plastic-Ltd; Lower Hutt, New Zealand) containing potting mix (AgResearch, Palmerston North, New Zealand) and grown under controlled conditions (day/night temperature 20 ± 2/15 ± 2°C, 50% relative humidity, 12 h light, 700 µmol m-2 s-1) in a plant growth chamber (Contherm BIOSYN Series Model 630).

**Figure 1:**
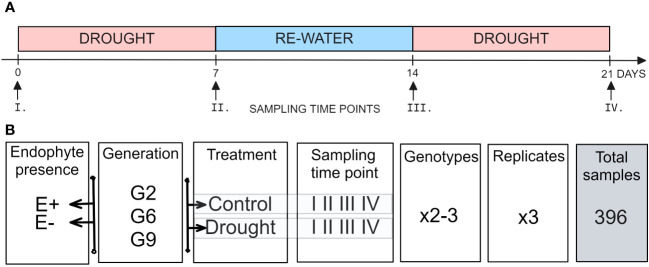
Setup of the experiment. **(A)** Temporal sampling points **(B)** experimental design.

For the drought stress experiment, plants were exposed to two treatments: (i) a control treatment, where plants were watered to saturation every other day, (ii) a drought stress treatment, where irrigation was withheld for seven days and then restored for seven days before another seven-day drought period. That way, the drought stress treatment was applied twice, each for seven days, with a week-long interval during which plants were watered to saturation daily (**Figure 1A**). Each treatment had three biological replicates, and the root trainers were rearranged weekly to account for spatial effects.

### 2.4 Harvesting of plant material

Pseudostem samples were collected at four time points: before the first drought treatment (I), after the first drought treatment (II), after rewatering (III) and after the second drought treatment (IV; **Figure 1A**). The samples were flash-frozen in liquid nitrogen and freeze-dried (SP Scientific, VirTis sentry 2.0 Freeze Dryer). Samples were collected in three replicates for each 2-3 genotypes, four time points (I, II, III, IV), two water availability treatments (control, drought), and two endophyte presence status (E-, E+) for each of the three plant generations (G2, G6, or G9; **Figure 1B**).

### 2.5 DNA extraction and epigenetic library preparation

Genomic DNA (gDNA) was extracted from freeze-dried plant tissue, homogenized using glass beads (Karl Hecht, 4 mm, 1 kg 41401004), with a modified CTAB protocol (Doyle and Doyle, 1987). The resulting pellets were dissolved in 100 μL of TE buffer (10 mM Tris-HCl; 0.1 mM EDTA) and treated with RNAse A (Thermo Fisher Scientific) at a 40 µg/mL concentration to remove RNA contamination. The quality of DNA samples was checked using NanoDrop 2000 (Thermo Fisher), and the concentration was determined using a PicoGreen Quant-It dsDNA assay (Invitrogen). All samples were diluted to 13.33 ng/μL in a volume of 100 μL.

Libraries were prepared according to an adapted protocol by van Gurp et al. (Van Gurp et al., 2016). Briefly, 400 ng of gDNA was digested with PacI and NsiI restriction endonucleases (New England BioLabs, Inc.), and barcoded adaptors were ligated to the fragments using T4 DNA ligase (New England BioLabs, Inc.). After ligation, samples were multiplexed (groups of 12), concentrated and purified with a PCR clean-up kit, followed by size-selection using 0.8x SPRIselect magnetic beads (Beckman Coulter, Inc.), and finally, nick translated with DNA polymerase I (New England BioLabs, Inc.) and 5-methylcytosine dNTP mix (Zymo Research). Nick-translated libraries were then treated with sodium bisulfite using the EZ DNA Methylation Lightning Kit (Zymo Research) to convert unmethylated (but not methylated) cytosines to uracil. The samples were PCR amplified with the EpiMark Hot Start Taq DNA Polymerase Kit (New England BioLabs, Inc.) and universal Illumina PE PCR primers. Another round of clean-up and size selection followed amplification. The quality and quantity of the libraries (> 2 nM) were checked using a PicoGreen dsDNA Assay Kit and qPCR, and the size profiles were determined using an Agilent Bioanalyzer and found to be 150-600 bp with a peak around 450 bp. Libraries were pooled with equimolar concentrations (each library consisted of 150 multiplexed samples) and sequenced on an Illumina HiSeq4000 (BGI, Hong Kong) in 2x150 bp paired-end mode over multiple lanes.

### 2.6 Identification of methylated cytosine and differentially methylated regions

For the analysis of methylated cytosines, pair-end reads of 150 bp were processed using the WellMeth pipeline (Malinowska et al., 2020) and mapped to the reference genome of *L. perenne* (Nagy et al., 2022), which contains 7 pseudo-chromosomes and 9400 unanchored scaffolds. The identification of methylated sites was followed by PCR duplicate removal. The methylation level of each cytosine covered by the analysis was calculated as a proportion of methylated cytosine (#C) to sequencing depth of a position (methylated and unmethylated #T) [#C/(#C + #T)]. The position-level methylation data were then filtered for a minimum of 3X read coverage.

To identify Differentially Methylated Regions (DMRs), we compared methylation levels of E+ and E- plants, as well as control and drought stressed plants using the WellMeth script, which implements a Hidden Markov Model-based framework adapted from the BisulFighter package (Saito et al., 2014). The DMRs were evaluated in all three sequence contexts (CG, CHG, CHH). To compare methylated regions within DMRs at the population level, consensus regions were called using the “*merge*” function from BEDtools (Quinlan and Hall, 2010). Regions with a minimum of five cytosines and frequency (number of methylated cytosines per length of the DMR) greater than 0.2 were retained for further analysis. To obtain a representative value for each region, we averaged the DNA methylation level over all genotypes and three biological replicates, resulting in 48 samples for further analysis (**Supplementary Figure S1**). These samples were then differentiated based on endophyte presence, plant generation, treatment, and time point.

### 2.7 Identification of repetitive DNA sequences

Transposable elements and repeats were identified using the RepeatMasker software tool (version open-4.0.6, Smit et al., 2013-2015) using the Liliopsida species model and RepBase Update 20160829. A custom pipeline was used to annotate the RepeatMasker output, as described in Malinowska et al. (2020). Briefly, it included three steps (i) assign RM class and target to each RepeatMasker hit, (ii) merge overlapping features of identical class and target categories, and (iii) produce separate BED format files for each RM class category to enable intersecting methylation features and RM features.

### 2.8 Genomic features

The “*intersect*” function from BEDtools (Quinlan and Hall, 2010) was used to identify specific chromosomal regions that overlapped with the positions and regions of interest that were previously identified as methylated positions and differentially methylated regions (DMRs) in the *L. perenne* genome. These identified regions were then compared to available genomic features, including repetitive DNA sequences, in the *L. perenne* genome.

### 2.9 Statistical analysis

We employed several statistical analyses to investigate the influence of generation and endophyte presence on methylation patterns. First, a Permutational Multivariate Analysis of Variance (PERMANOVA) was conducted using the ‘*adonis2*’ function from the ‘*vegan*’ package (Oksanen et al., 2022). This non-parametric test utilized a Bray-Curtis dissimilarity matrix to assess the differences in methylation patterns between samples. The model included plant generation, endophyte presence status, and their interaction as independent variables, with significance determined through permutations.

Pairwise comparisons were performed using the ‘*pairwiseAdonis*’ package (Martinez Arbizu, 2020) to examine dissimilarities between groups, considering the combination of plant generation and endophyte presence/absence.

A Principal Component Analysis (PCA) was also conducted using the ‘*prcomp*’ function in R, with the Bray-Curtis dissimilarity matrix as input. This analysis provided insights into the underlying structure and grouping of the DNA methylation data.

To investigate the impact of the treatment variable on DNA methylation, we used the ‘*rstatix*’ package in R (Kassambara, 2023). First, we conducted an analysis of variance with covariate adjustment (ANCOVA), regressing DNA methylation on “sampling time” and the treatment variable (control and drought) within specific groups defined by generation and endophyte presence. We fitted a general linear model to examine the covariates’ influence on DNA methylation across groups. Pairwise comparisons using the “*emmeans*” test were performed to analyze treatment differences within each group, considering sampling time. We adjusted p-values for multiple comparisons using the Bonferroni method.

## 3 Results

### 3.1 At the global methylation level, the majority of the methylated cytosines identified were in the CHH context

The epiGBS method utilized in this study allowed for the analysis of methylated patterns in the subset of *L. perenne* genome under artificial association with the fungal endophyte AR37 and under drought stress. After excluding samples with low coverage, a total of 392 samples were included in the global methylation analysis (135 for G2, 141 for G6, and 116 for G9) as listed in **Figure 1B**. In the analyzed population, a diverse range of methylated positions was identified, with 11.4 million unique methylated sites. These unique sites represent distinct positions identified at least once in the dataset. The total number of methylated sites, including each unique site’s frequency across all samples, amounted to 244 million. Specifically, 61.9 million sites were fully methylated (100%), while 6.8 million sites remained unmethylated (0%) (**Supplementary Table S1**).

**Figure 2:**
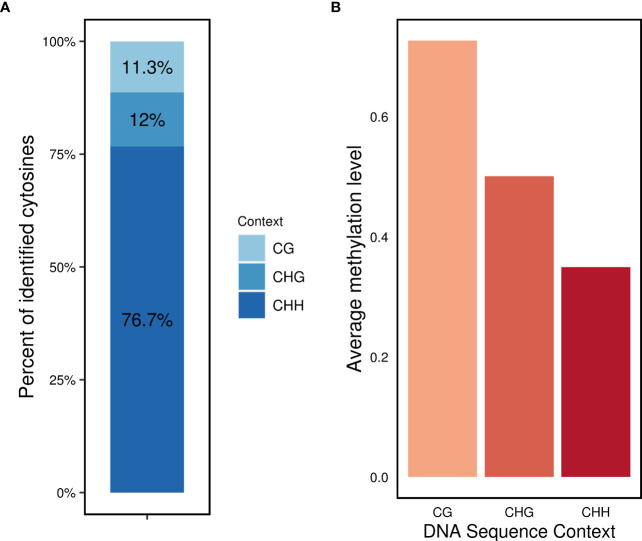
DNA methylation profile in *L. perenne* **(A)** relative proportion of methylated cytosines in the three sequence contexts. **(B)** Global average DNA methylation of CG, CHG and CHH in *L. perenne*.

Regarding the distribution of methylated cytosines in different sequence contexts, the CHH context accounted for the highest number of methylated sites (76.7%), followed by the CHG (12%) and CG (11.3%) contexts (**Figure 2A**). The average methylation levels were highest in the CG context (0.72), followed by the CHG context (0.5), and lowest in the CHH context (0.35) (**Figure 2B**).

**Figure 3:**
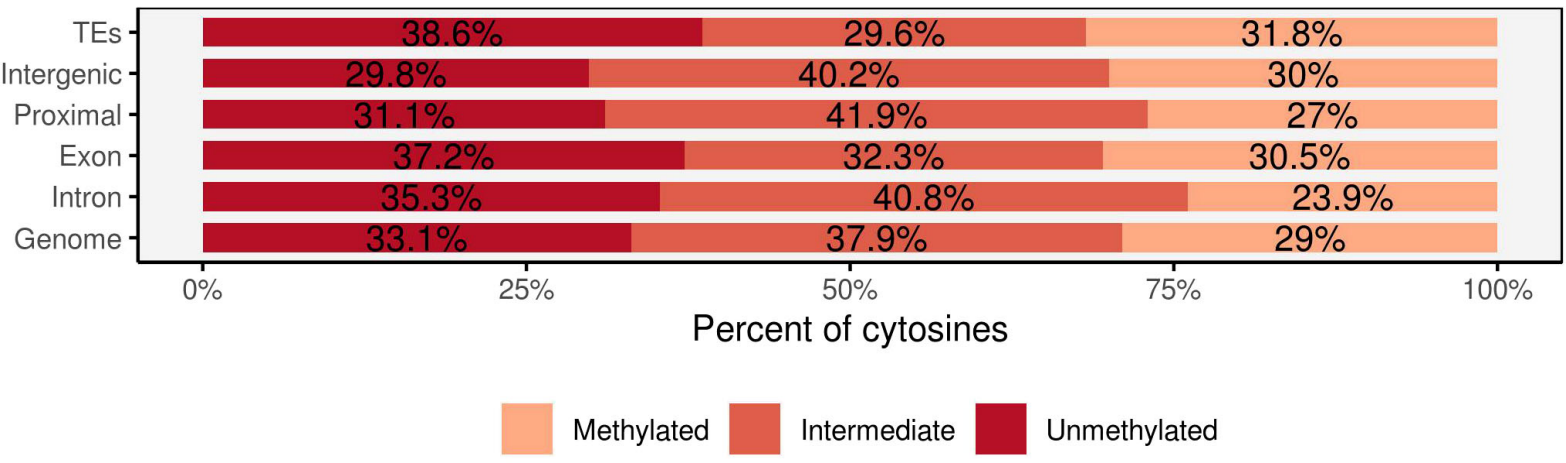
The percentage of methylated cytosines in the four main genomic regions: transposable elements (TEs), intergenic, genic (intron, exon), regions proximal to genes and the overall covered region. Each methylated position was classified into three methylation levels: unmethylated (<10%), intermediate (>10% but <90%), and methylated (>90%).

Analysis of the genomic features of methylated sites revealed that 35% of methylated cytosines were found in intergenic regions (between 5–100 kb upstream of genes), while 14.5% were in gene bodies (including introns and exons). Proximal regions to genes (up to 5 kb upstream of transcription start sites and up to 2 kb after transcription end sites) accounted for 27% of methylated sites, and 23% were located in transposable elements (TEs). Both transposable elements and exons exhibited intermediate methylation levels of 29.6% and 32.3%, respectively, with methylated cytosines accounting for 31.8% in TEs and 30.5% in exons (**Figure 3**). Intergenic regions and proximal regions to genes demonstrated comparable methylation patterns, with a higher proportion of intermediate methylation (40.2% in intergenic regions and 41.9% in proximal regions). Intron regions stood out with a lower ratio of methylated cytosines (23.9%) and higher proportions of unmethylated cytosines (35.3%).

### 3.2 Differentially methylated regions are mainly found in the intergenic regions

We conducted differential methylation analysis on ryegrass samples to explore the changes in methylation patterns in response to the presence of *Epichloë*. Our analysis identified over 28,000 unique DMRs across all sample pairs (**Supplementary Figure S2**). In addition, all DMRs were distributed throughout the ryegrass genome with no positional enrichment or bias (**Supplementary Figure S2**).

**Figure 4:**
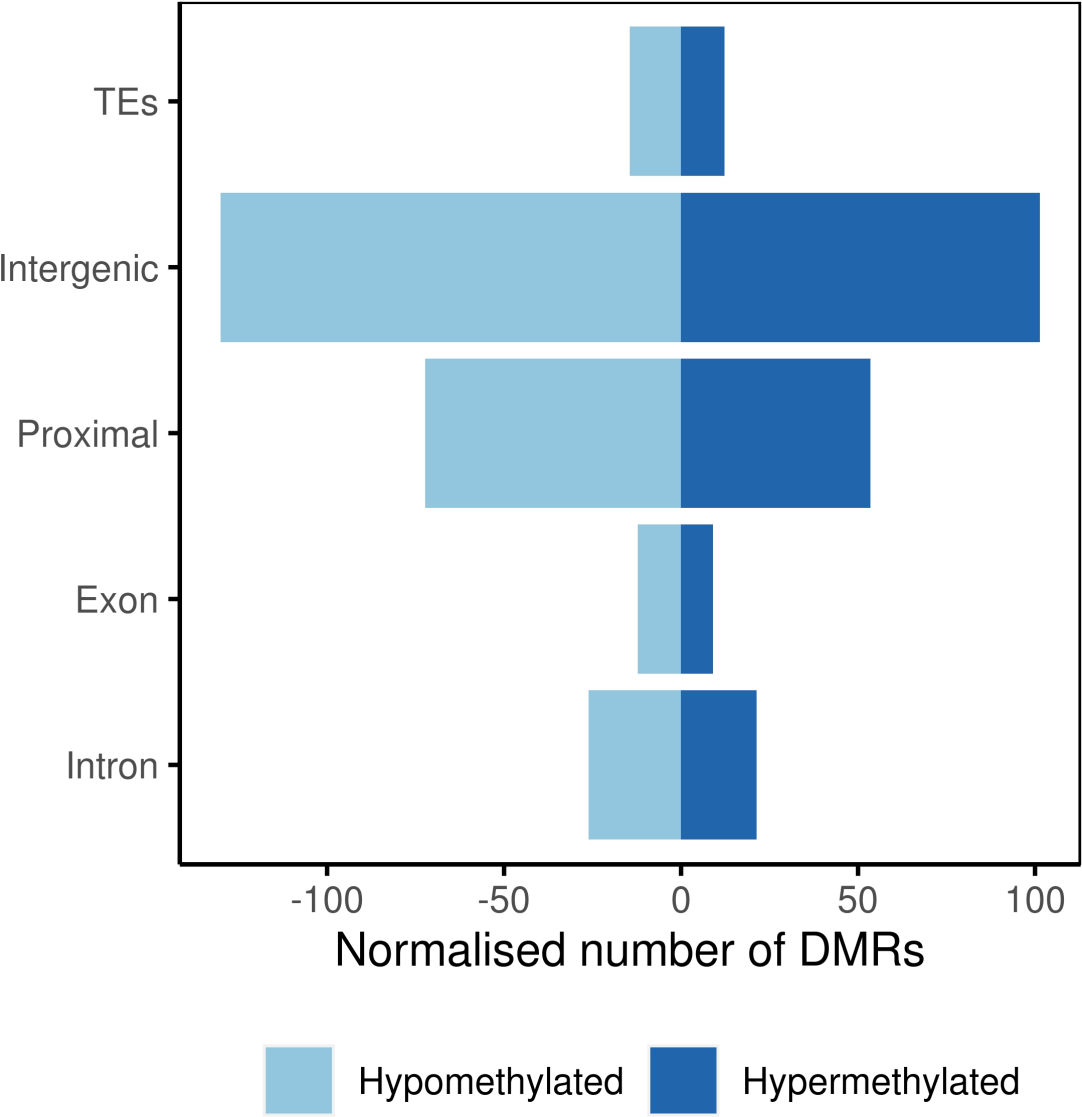
Number of differentially methylated regions in genomic features.

At the population level, majority of the DMRs were located in intergenic regions followed by proximal regions and repeats, retrotransposons, and transposons, all grouped under the term ‘transposable element region’ (TEs; **Figure 4**). Genic regions, including exons and introns, showed lower representation among all the analyzed genomic features.

The context of methylation also influenced the genomic localization of these DMRs (**Table 1**). Proximal regions exhibited a higher prevalence of DMRs in the CG context. In the CHG context, both introns and exons displayed slightly elevated proportions of DMRs. Notably, both CG and CHG contexts demonstrated a greater concentration of DMRs within or near genes compared to the CHH context. Proportionally, the majority of DMRs within the CHH context were found in intergenic regions and transposable elements, while exonic regions exhibited the lowest DMR frequency.

**Table 1:** Proportion of DMR in genetic features for each methylation context.

	CG	CHG	CHH
**Exon**	0.11 (n = 244)	0.13 (n = 356)	0.08 (n = 494)
**Intron**	0.17 (n = 380)	0.21 (n = 590)	0.15 (n = 950)
**Proximal**	0.26 (n = 599)	0.23 (n = 636)	0.22 (n = 1423)
**Intergenic**	0.23 (n = 518)	0.22 (n = 601)	0.25 (n = 1607)
**TEs**	0.24 (n = 549)	0.21 (n = 592)	0.31 (n = 2006)

We applied a series of filtering criteria to the initial 28,000 unique DMRs to identify a robust set of differentially methylated regions. We categorized these regions as rare, consistent, or highly represented sets. In total, we identified over 15,000 rare DMRs that were present in less than 50% of the analyzed samples. Additionally, 12,674 regions with altered methylation levels were present in at least 50% of the samples and were classified as consistent DMRs. Of these consistent DMRs, 2,196 highly represented regions were identified in all analyzed samples.

### 3.3 The presence of the endophyte affects methylation profile differently across generations

We performed PERMANOVA and PCA analyses on a Bray-Curtis dissimilarity matrix constructed from 115,595 methylation loci to assess DNA methylation differences within the population. Before analysis, a filtering process was employed to exclude loci with missing values (NAs) and low variance, ensuring high-quality and informative methylation loci inclusion.

The PERMANOVA analysis (**Table 2**) indicated highly significant variations in the dissimilarity matrix influenced by generation, endophyte presence, and their interaction. Generation demonstrated a significant effect, explaining 10.9% of the observed variation (R2 = 0.10896, F = 3.4745, p < 0.001). Similarly, endophyte presence exhibited a significant effect, explaining 13.3% of the variation (R2 = 0.13256, F = 8.4541, p < 0.001). The interaction between these factors also yielded a significant effect, explaining 10% of the variation (R2 = 0.09992, F = 3.1861, p < 0.001).

**Table 2:** PERMANOVA Partitioning and Analysis of DNA Methylation patterns.

	Df	SS	R2	F	Pr(>F)
**generation**	2	0.034	0.109	3.475	0.0001
**endophyte**	1	0.042	0.133	8.454	0.0001
**generation:endophyte**	2	0.032	0.100	3.186	0.0001
**Residual**	42	0.208	0.659		
**Total**	47	0.316	1.000		

Df, degrees of freedom; SS, Sum of Squares; R2, proportion of variation in the dissimilarity matrix; F, F-statistic, Pr(>F), p-value.

**Figure 5:**
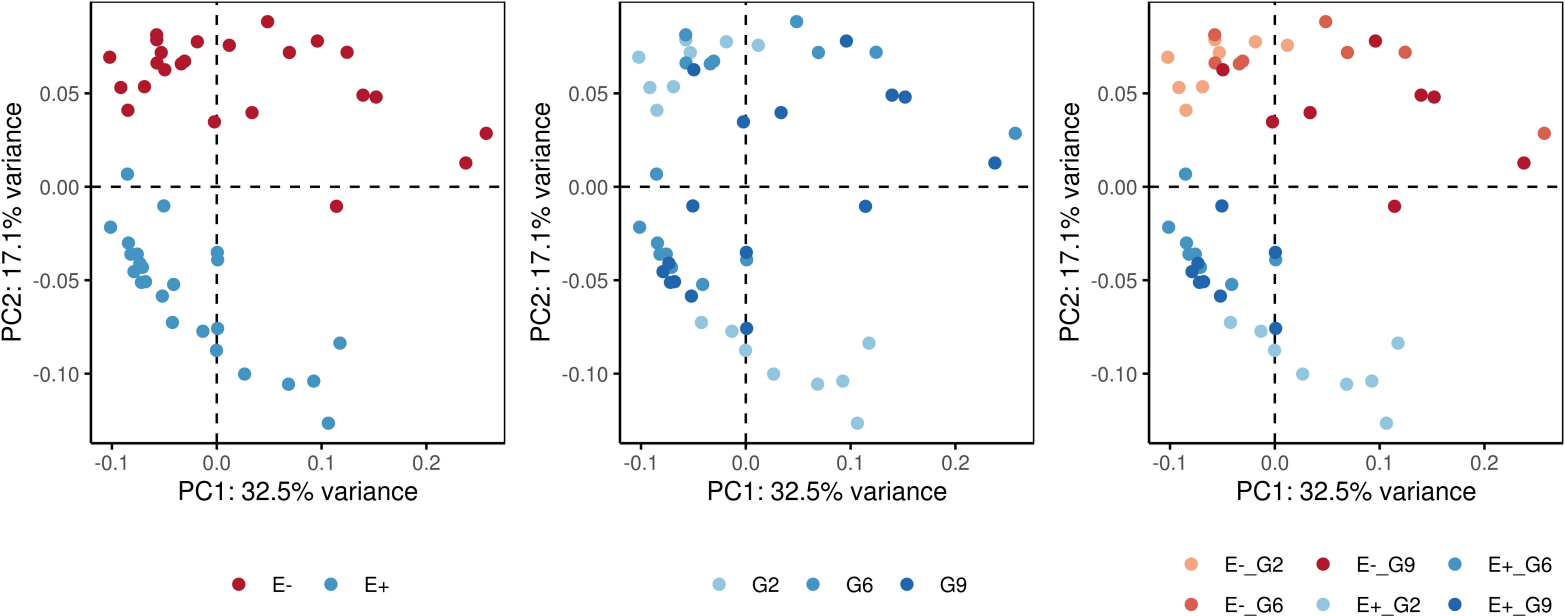
Principal component analysis (PCA) plots showing the dissimilarity-based distances of samples for the measurement of DNA methylation. Colors represent different factors: endophyte presence (E+) or absence (E-), generations (G2: generation 2; G6: generation 6; G9: generation 9), and a combination of endophyte status and plant generation.

Pairwise PERMANOVA comparisons (**Table 3**) revealed significant differences among the E-_G2, E-_G6, E-_G9, E+_G2, E+_G6, and E+_G9 groups. The dissimilarity observed were primarily concentrated along the first two principal components, which accounted for 49.6% of the variance (**Figure 5**). Notably, PC2 exhibited a clear separation between E+ and E- groups, while PC1 and PC2 demonstrated subtler differences between generations within both E+ and E- groups. These results emphasise the contributions of generation and endophyte presence to the observed variations in DNA methylation patterns, reflecting the combined influence of genetic (generation) and environmental factors (endophyte presence).

**Table 3:** Pairwise PERMANOVA of global DNA methylation patterns between groups based on endophyte presence and generations F-statistics values are below diagonal.

	E-_G2	E+_G2	E-_G6	E+_G6	E-_G9	E+_G9
**E-_G2**		**0.015**	**0.015**	**0.015**	**0.015**	**0.030**
**E+_G2**	4.666		**0.015**	**0.015**	**0.015**	**0.015**
**E-_G6**	2.933	5.077		**0.030**	**0.015**	**0.015**
**E+_G6**	5.625	4.253	5.528		**0.015**	**0.015**
**E-_G9**	2.683	4.337	2.051	4.927		**0.015**
**E+_G9**	5.419	4.015	5.739	4.742	4.715	

Adjusted p-values (Bonferroni multiple comparison correction) are above the diagonal. Values of p<0.05 (in bold) are considered significant.

### 3.4 E+ plants are consistently hypomethylated

**Figure 6:**
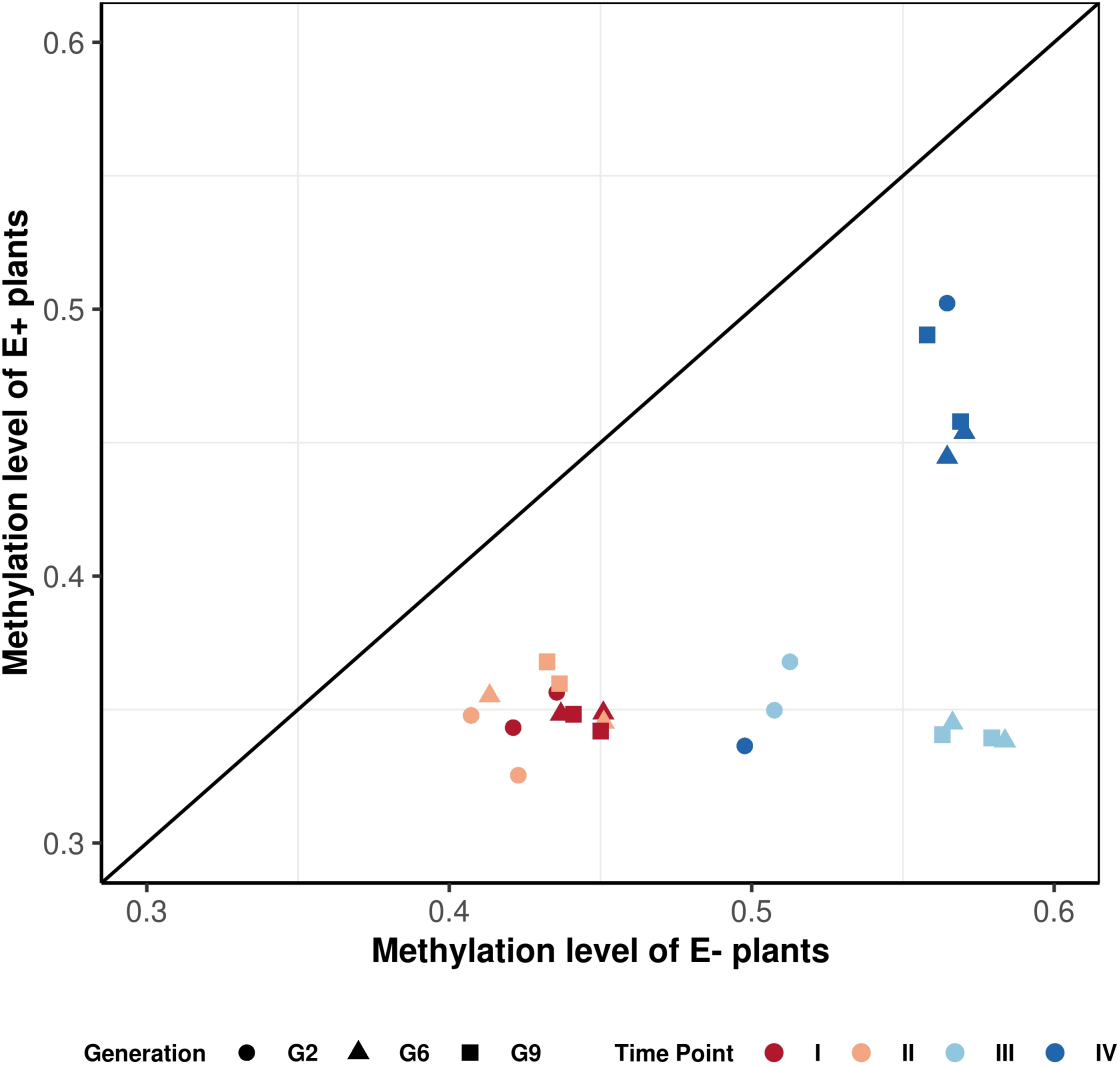
Correlation plot of methylation patterns observed over consistent DMRs. Each point represents a difference in methylation between E- and E+ plants for each generation (represented by different shapes), treatment and time point of sampling (represented by a different color). The points are averaged over three replicates. There are two data points for each color-shape combination, and they represent control and drought treatment accordingly. The graph shows that the points for the endophyte-infected plants are located below the diagonal line, indicating global hypomethylation.

To investigate the overall impact of endophyte presence on host methylation, we compared the average methylation levels in E+ and E- samples across different generations, water availability treatments, and sampling time points (**Figure 6**). We averaged the samples over biological replicates and only considered the methylation of 12,674 consistent DMRs.

**Figure 6** shows that E+ plants have consistently lower methylation levels compared to E- plants within the same generation and grown under the same treatment. The hypomethylation level in E+ plants was associated with various factors, including the sampling time point (connected to both treatment and growth stage), generation, and water availability treatment. All generations showed similar hypomethylation levels for time point I and II (14-18%; **Table 4**). Time point IV was distinct from early sampling time, but the absolute methylation change was still low to moderate, similar to time points I and II. The difference in methylation levels between E+ and E- plants was highest at time point III (after plants had been rewatered following the first drought treatment), reaching 28% for G6 and G9 (**Table 4**). In comparison, the presence of endophytes only lowered the overall methylation in consistent DMRs by around 20% in G2, which follows the pattern shown in **Figure 6** and distinguishes it from G6 and G9.

**Table 4:** Absolute change in methylation of consistent DMRs between endophyte-negative and E+ samples.

Generation	Treatment	Time Point			
		I	II	III	IV
**G2**	Control	-0.14	-0.17	-0.20	-0.05
**G2**	Drought	-0.14	-0.11	-0.21	-0.21
**G6**	Control	-0.14	-0.12	-0.28	-0.13
**G6**	Drought	-0.18	-0.17	-0.27	-0.12
**G9**	Control	-0.16	-0.14	-0.28	-0.12
**G9**	Drought	-0.15	-0.14	-0.27	-0.07

The effect of the endophyte presence was significant for each pair, as estimated with TukeyHSD (p < 0.001).

Our study also investigated the impact of endophyte presence on methylation in different contexts, specifically CG, CHG, and CHH methylation (**Supplementary Table S2**). Interestingly we did not observe any significant differences based on the context. The absolute change in methylation levels was consistently observed across all contexts, with no apparent pattern or bias for a specific context.

### 3.5 A contrasting methylation level was observed between E+ and E- plants

**Figure 7:**
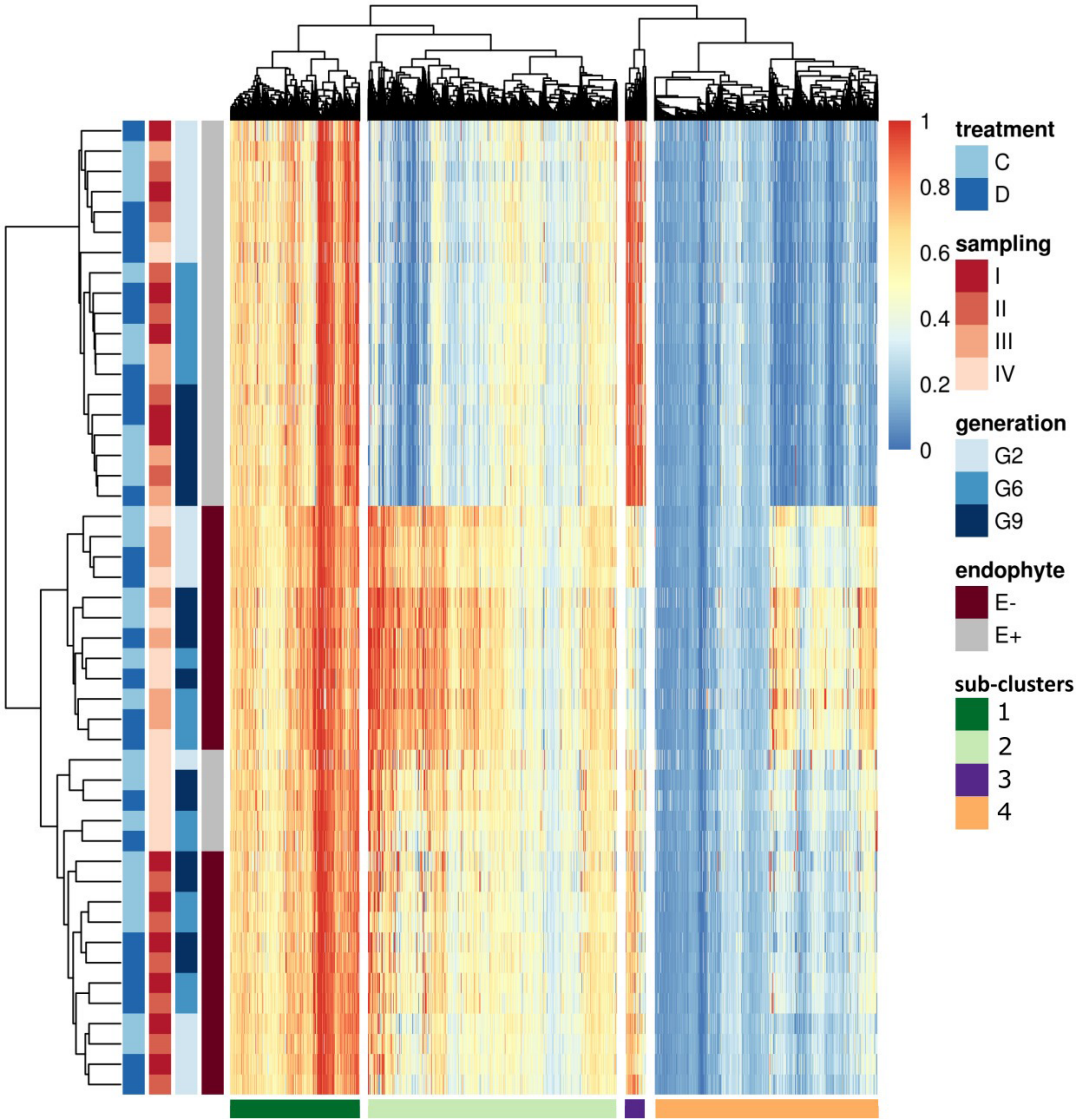
Heatmap of all the consistent DMRs. On the top of the heatmap hierarchical tree clusters DMRs (divides samples in four sub-clusters), and on the left side, samples are clustered based on endophyte presence (E-: endophyte negative, E+: endophyte positive), generation, the time point of the sampling and applied water treatment (C: control, D: drought). The darkest blue indicated the lowest methylation (0) in the color key panel, while red indicated the highest methylation level (1).

To understand the variation in methylation patterns and identify differential methylation under biotic and abiotic stimuli, we compared consistent DMRs (12,674) and performed hierarchical clustering (**Figure 7**). Consistent with **Figure 5**, the most significant differences were observed between E+ and E- plants. Within E+ plants, methylation patterns were also associated with plant generation and, with the exception of G2 samples at time point IV, water treatment and sampling time were of secondary importance. In the absence of endophyte, plant generation played a secondary role, and methylation patterns seemed to be more closely connected to the sampling time and, to some extent, applied water treatment. In the E- plants, two early sampling points showed different methylation patterns compared to time points III and IV; within those, applied water availability treatment also appeared to affect the methylation of the analysed DMRs. Finally, only G2 seemed to separate from the two later generations in the E- plants.

Hierarchical clustering identified four distinct sub-clusters based on the methylation patterns within the dataset. (**Figure 7**). We investigated the effects of drought treatment (control and drought) and sampling time point on methylation levels within each sub-cluster and group based on endophyte status and plant generation.

Significant treatment effects were observed in sub-cluster 1 (2635 DMRs) for several groups (E-_G2, E-_G6, and E-_G9). Sub-cluster 2 (5077 DMRs) exhibited significant effects of both sampling time points and applied treatment across all groups. Sub-cluster 3 (415 DMRs) and sub-cluster 4 (4546 DMRs) showed significant effects of sampling time points for all groups, while the effect of treatment varied across different groups (**Supplementary Table S3**).

Effect sizes indicated small to moderate influences in sub-clusters 1 and 2, while sub-cluster 3 displayed a mix of effect sizes ranging from 0.0000124 to 0.102. Sub-cluster 4 exhibited larger effect sizes, indicating a stronger relationship between the factors and DNA methylation (**Supplementary Table S3**).

**Figure 8:**
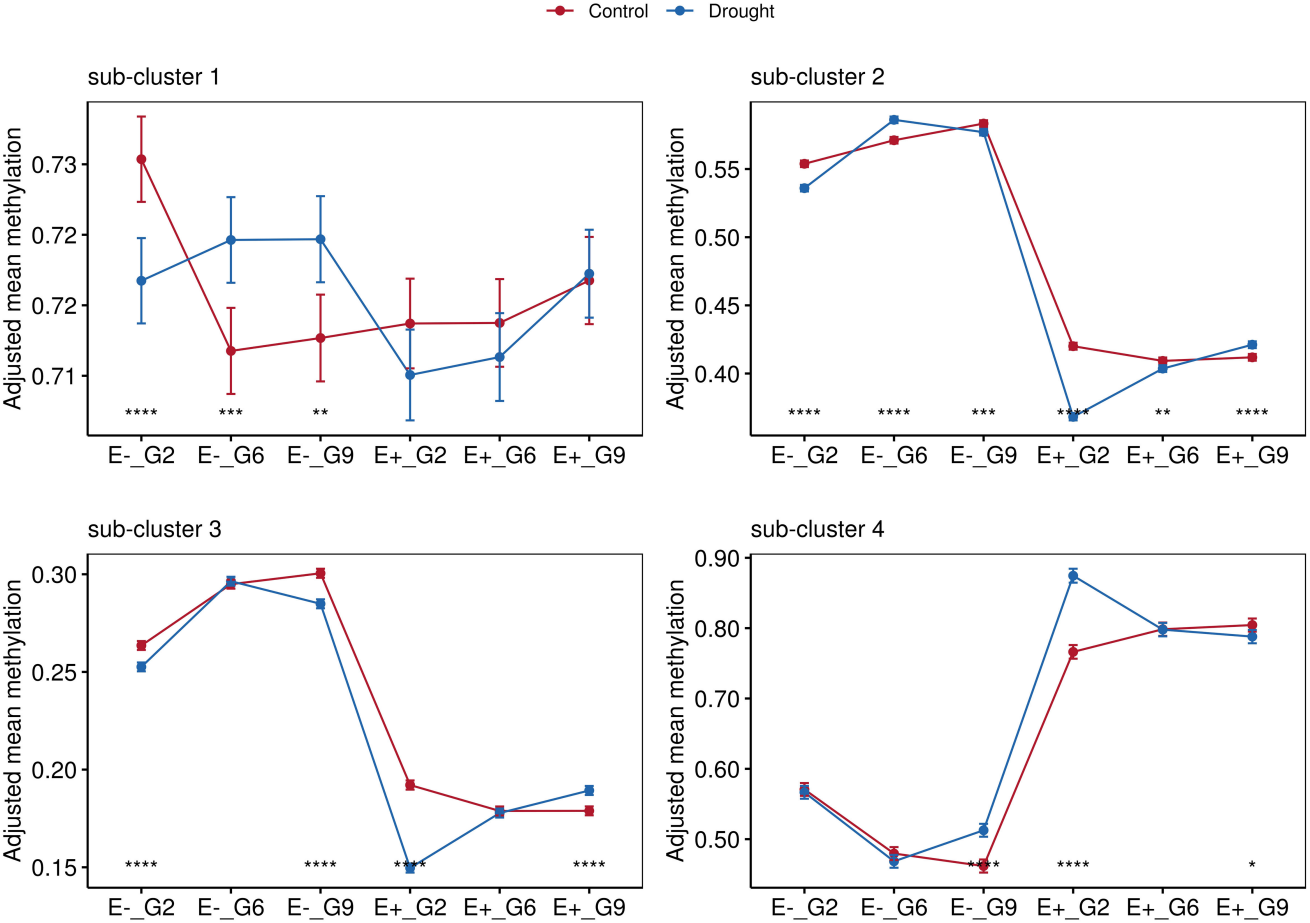
Comparisons between treatments for groups according to endophyte presence (E-, E+) and generation (G2, G6 and G9). Data points represent mean values with standard error bars. Statistical significance indicated by asterisks (*). Non-significant comparisons (ns) not shown. Four facets represent the average methylations of the three sub-clusters identified in **Figure 7**.

Pairwise comparisons using analysis of covariance (ANCOVA), with sampling time points controlled as covariates, were conducted to explore these relationships further. Significant differences between the control and drought groups were observed in sub-cluster 1 exclusively for the E- plants. Consistent with the findings in **Figure 6**, hypomethylation in E+ plants was observed in sub-cluster 2 and sub-cluster 3 (**Figures 6**, **7**). Sub-cluster 2 showed significant differences between the control and drought groups for E+ and E- plants across all generations. In sub-cluster 3, applied treatment had a significant effect for E+_G2, E+_G9, E-_G2, and E-_G9. Lastly, in sub-cluster 4, E+ plants exhibited an overall higher methylation level than E- plants (**Figure 8**). Significant differences between the control and drought groups were also observed for E+_G2, E+_G9, and E-_G9.

Analysis of DMR locations within each sub-cluster revealed a varied distribution across genomic features (**Table 5**). Sub-cluster 1 exhibited a higher proportion of DMRs in proximal and intergenic regions with fewer in genic locations. Sub-cluster 2 had a pronounced distribution of DMRs in genic (exons and introns) and proximal regions, which could potentially be linked to gene expression regulation. Sub-clusters 3 and 4 displayed a substantial proportion of DMRs in transposable elements and non-coding intergenic regions. This distribution underscore a potential role of these sub-clusters in genome stability and possibly trans-regulation of gene expression.

**Table 5:** Proportion of DMRs across genomic features for each sub-cluster identified in **Figure 6**.

	sub-cluster 1	sub-cluster 2	sub-cluster 3	sub-cluster 4
**Exon**	0.05	0.08	0.07	0.05
**Intron**	0.10	0.17	0.15	0.10
**Proximal**	0.24	0.26	0.18	0.20
**Intergenic**	0.31	0.27	0.19	0.28
**Tes**	0.30	0.22	0.40	0.37

The analysis revealed diverse effects of drought treatment and sampling time point on methylation levels across sub-clusters, highlighting the complex interplay between environmental factors and epigenetic patterns. Significant interactions were observed between endophyte presence, plant generation, and the effects of drought treatment on methylation patterns, underscoring the role of these grouping variables in shaping the response to environmental stressors.

## 4 Discussion

The present study examines the influence of the *Epichloë* sp. *Lp*TG-3 strain AR37 on the proportion of the methylome of its host plant, *Lolium perenne*, under drought stress conditions using epiGBS. Endophytic fungi have been shown to influence methylation patterns of host plants, suggesting potential benefits to plant fitness and response to stress (Hubbard et al., 2014; Gupta et al., 2020). To the best of our knowledge, this is the first investigation of the effects of the *Epichloë* sp. *Lp*TG-3 strain AR37 on the methylome of *L. perenne* over an extended period of a breeding program. Since un-inoculated *L. perenne* plants were not included in this study, we employed a comparative approach within each generation, using the endophyte-negative (E-) individuals as a baseline for comparison. This allows us to examine the changes in the methylome over time and under drought stress conditions associated with the presence of the endophyte. By analysing three selected plant generations from a nine-year seed maintenance program, we have gained valuable insights into the dynamic changes in the methylome associated with the presence of the endophyte in *L. perenne*.

### 4.1 Endophyte presence is associated with hypomethylation in its *Lolium perenne* host

Epigenetic modulations play an important role in plant-microbe interactions (Alonso et al., 2019; Ramos-Cruz et al., 2021; Hannan Parker et al., 2022). Studies have shown that epigenetic mechanisms, such as methylation, regulate response networks and may affect genome reorganization (Boyko and Kovalchuk, 2008). Specifically, plant-microbe interactions, including those with fungi and bacteria, can lead to changes in the host plant’s methylation patterns, linking epigenetics to the regulation of these interactions (Stroud et al., 2013; Rambani et al., 2015; Varga and Soulsbury, 2017; Geng et al., 2019; Atighi et al., 2020). We found a significant decrease in DNA methylation in the E+ individuals across three generations and under different water treatments, compared to the E- individuals within the same generation. This finding aligns with prior studies demonstrating hypomethylation in response to interactions between plants and microorganisms. This suggests that the loss of methylation can promote infections by both mutualistic organisms (Satgé et al., 2016; Niyikiza et al., 2020) or pathogens (Pavet et al., 2006; Akimoto et al., 2007; Yu et al., 2013; Hewezi et al., 2017; Geng et al., 2019; Catoni et al., 2022). However, several studies have documented opposing results, such as the critical role of CHH hypermethylation in nodule formation in *Medicago truncatula* (Pecrix et al., 2022) and elevated DNA methylation levels in response to arbuscular mycorrhizal fungi colonisation in *Geranium robertianum* (Varga and Soulsbury, 2019). These and other similar studies suggest that the impact of hypo- or hypermethylation following infection is context dependent and often highlights the predominant role of CHH methylation (Geng et al., 2019; Atighi et al., 2020; Xiao et al., 2021).

In our experiment, we detected a large number of DMRs, both hyper- and hypomethylated, in response to infection with endophyte (**Figure 4**). Most of the DMRs were also identified in the CHH context (**Supplementary Table S2**), which aligns with the observation that over 70% of identified methylated cytosines were in the CHH context (**Figure 2**). This is in agreement with previous studies that reported similar CHH methylation changes in response to pathogens (Pavet et al., 2006; Dowen et al., 2012; Geng et al., 2019; Catoni et al., 2022). However, an interesting finding of our study is that we did not observe any significant differences in the degree of hypomethylation based on the sequence context, such as CG, CHG or CHH methylation, but it is consistently observed across all contexts (**Supplementary Table S2**). These results raise important questions about the mechanisms by which *Epichloë* sp. leads to hypomethylation in its grass host. Our findings highlight the complexity of the analysed associations and the need for further research to fully understand the mechanisms and extent to which endophytic fungi can influence plant methylation (Pélissier et al., 1999; Zhu, 2009; Law and Jacobsen, 2010; Dowen et al., 2012; Gent et al., 2013; Zemach et al., 2013; Dupont et al., 2015; Satgé et al., 2016; Schmid et al., 2017; Atighi et al., 2020; Kou et al., 2021).

### 4.2 Generation effect on methylation

In this study, we investigated the relationship between the endophyte and DNA methylation patterns in different generations of *L. perenne* plants. Our findings suggest that the observed DNA methylation patterns may be influenced by the duration of the endophyte-plant association and the accumulation of genetic and epigenetic changes over time. The global DNA hypomethylation pattern observed across generations cannot be solely attributed to the presence of the endophyte, AR37. Instead, our analysis revealed a significant interaction between endophyte presence and generation, indicating a complex interplay between these factors (**Table 2**). The genetic background of the plant and the influence of the endophyte are both crucial in shaping DNA methylation patterns across generations (Madlung and Comai, 2004).

In our previous study (Forte et al., 2020), we found a decrease in fungal biomass in the G9 generation of endophyte-infected plants without a corresponding reduction in fungal-induced gene expression in the host plant. In the current study, we observed that changes in fungal biomass (based on the previous work) did not directly correlate with changes in differential methylation levels. While the E+ samples were grouped based on generation (G2 vs G6 and G9), the distinction of E- plants based solely on generation was not evident, suggesting potential intricacies in the association between generation, endophyte presence, and DNA methylation patterns.

The multiple-generation aspect of this experiment raises the possibility of a genetic component underlying the changes in methylation patterns. As previous studies have shown, epigenetic modifications, such as DNA methylation, can be passed on to future generations, providing a potential mechanism for how endophyte-host interactions shape the outcome over time (Luna and Ton, 2012; Henry et al., 2013; Rendina González et al., 2018; Gallusci et al., 2022). The distinct methylation patterns observed across generations suggest that the host plant may undergo genetic and epigenetic changes that improve the compatibility of the interaction with the endophyte over time. However, it is still unclear whether the changes observed in the host plant are due to passive influences exerted by the endophyte on plant cells (Atighi et al., 2020) or if the endophyte actively modulates the host’s epigenetic machinery to facilitate its persistence within the host (Dalakouras et al., 2022)

It is important to note that the un-inoculated *L. perenne* plants were not included in this study, and therefore, the contribution of the “naïve” epigenome to the plant’s response to primary infection remains unclear. Nonetheless, our observations of significant hypomethylation in E+ populations over generations, when compared to E- populations, suggest the involvement of the endophyte in the observed epigenetic changes. Given that the endophyte-free plants used in this study were derived from plants that lost their endophyte during seed propagation, caution is advised when using them as controls for endophyte effects. The loss of endophytes may not occur randomly, and genetic or epigenetic changes between generations could increase the likelihood of endophyte loss. Furthermore, it is important to note that E+ plants represent a continuum across all generations, while there is a discontinuity in E- material across generations. Thus, when evaluating the relationship between “internally generated” E- material and E+ material, these differences should be taken into account.

### 4.3 *Epichloë* sp. LpTG-3 strain AR37 affects methylation pattern under drought stress

We aimed to investigate the influence of the fungal endophyte *Epichloë* sp. *Lp*TG-3 strain AR37 on DNA methylation patterns in response to drought stress in *L. perenne*. Our results reveal a significant impact of endophyte presence on DNA methylation in response to applied drought stress. E+ plants exhibited distinctive methylation profiles compared to E- plants, providing evidence for endophyte-mediated modulation of DNA methylation. Moreover, the distribution patterns of DMRs across genomic features within the identified sub-clusters shed light on the connections between environmental cues, endophyte interaction, and epigenetic adaptations, highlighting the complexity of plant responses to combined biotic and abiotic stressors.

The findings of our study contribute to the growing body of research on the effects of fungal endophytes on plant responses to abiotic stress, particularly drought. Previous studies have reported the beneficial effects of fungal endophytes in enhancing the adaptive capabilities of their host plants in response to abiotic stress, including drought (Hubbard et al., 2014; Kumari and Vujanovic, 2020; Hosseyni Moghaddam et al., 2021). However, the precise role of *Epichloë* spp. in conferring drought tolerance to its grass hosts remains a subject of debate (Hume et al., 2016; He et al., 2017; Schmid et al., 2017; Xu et al., 2017).

Our study highlights the potential role of DNA methylation patterns as one mechanism through which endophytes influence plant responses to drought stress. However, it is important to note that our findings do not provide a conclusive assessment of whether the observed modulation of DNA methylation is beneficial or detrimental to drought tolerance in *L. perenne*. Further investigations incorporating physiological data and larger datasets are necessary to elucidate the specific contributions of our findings to drought tolerance in this plant species.

Further research is warranted to comprehensively understand the interactions between endophytes, DNA methylation patterns, and drought tolerance in *L. perenne*. Such studies should integrate physiological information and incorporate broader datasets to provide a more comprehensive understanding of the potential benefits or complexities associated with endophyte-mediated DNA methylation modulation in drought stress.

## Data availability statement

The original contributions presented in the study are included in the article/**Supplementary Files**, further inquiries can be directed to the corresponding author.

## Author contributions

MM: Formal Analysis, Investigation, Visualization, Writing – original draft, Writing – review & editing. FF: Conceptualization, Data curation, Methodology, Writing – original draft. IN: Data curation, Writing – review & editing. JS: Conceptualization, Methodology, Supervision, Writing – review & editing. PD: Conceptualization, Supervision, Writing – review & editing, Project administration. DH: Resources, Writing – review & editing. RJ: Resources, Writing – review & editing. WS: Resources, Writing – review & editing. TA: Conceptualization, Funding acquisition, Project administration, Supervision, Writing – review & editing.
