# Corrigendum: Methylome changes in *Lolium perenne* associated with long-term colonisation by the endophytic fungus *Epichloë* sp. *Lp*TG-3 strain AR37

**DOI:** 10.3389/fpls.2023.1332690

**Published:** 2023-11-13

**Authors:** Flavia Pilar Forte, Marta Malinowska, Istvan Nagy, Jan Schmid, Paul Dijkwel, David E. Hume, Richard D. Johnson, Wayne R. Simpson, Torben Asp

**Affiliations:** ^1^ Center for Quantitative Genetics and Genomics, Faculty of Technical Sciences, Aarhus University, Aarhus, Denmark; ^2^ Ferguson Street Laboratories, Palmerston North, New Zealand; ^3^ School of Fundamental Sciences, Massey University, Palmerston North, New Zealand; ^4^ AgResearch, Grasslands Research Centre, Palmerston North, New Zealand

**Keywords:** *Lolium perenne*, *Epichloë* sp., DNA methylation, endophytic fungi, drought stress, plant-microbe interactions, artificial association, generation effect

In the published article, there was an error in **Figure 8**. The Figure comprises four smaller plots, each representing a distinct sub-cluster. However, sub-clusters 2 and 3 erroneously duplicated the content of sub-cluster 1. The corrected **Figure 8** and its caption appear below.

**Figure 8:**
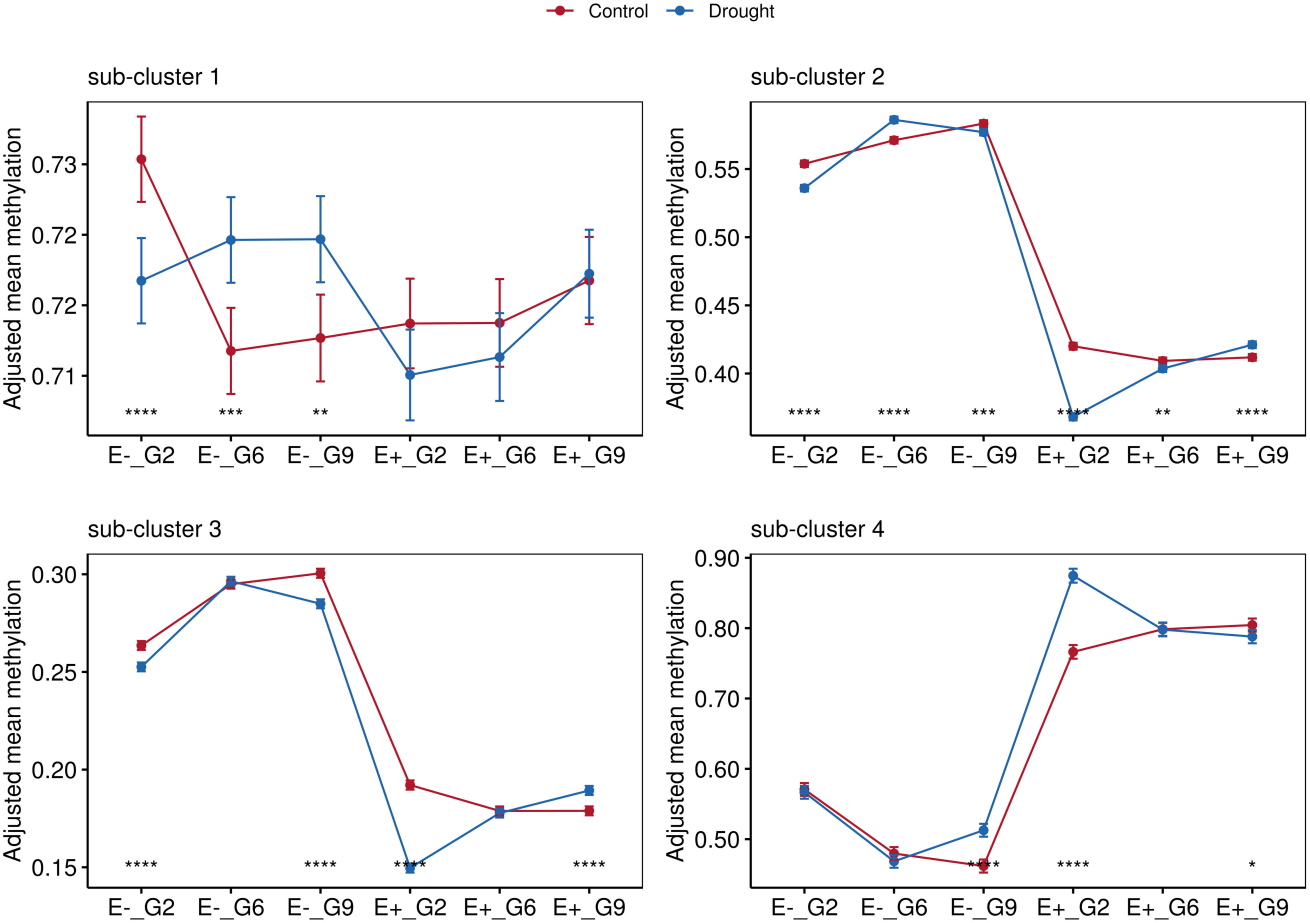
Comparisons between treatments for groups according to endophyte presence (E-, E+) and generation (G2, G6 and G9). Data points represent mean values with standard error bars. Statistical significance indicated by asterisks (*). Non-significant comparisons (ns) not shown. Four facets represent the average methylations of the three sub-clusters identified in Figure 7.

The authors apologize for this error and state that this does not change the scientific conclusions of the article in any way. The original article has been updated.

