# Supplementary material for: Methylome changes in Lolium perenne associated with long-term colonisation by the endophytic fungus Epichloë sp. LpTG-3 strain AR37
Source: Front Plant Sci. 2023 Sep 22;14:1258100. doi: 10.3389/fpls.2023.1258100 (PMC10557135; doi:10.3389/fpls.2023.1258100)
Supplement: Supplementary file 1 [file DataSheet_1.docx]

Supplementary Material

# Supplementary Figures


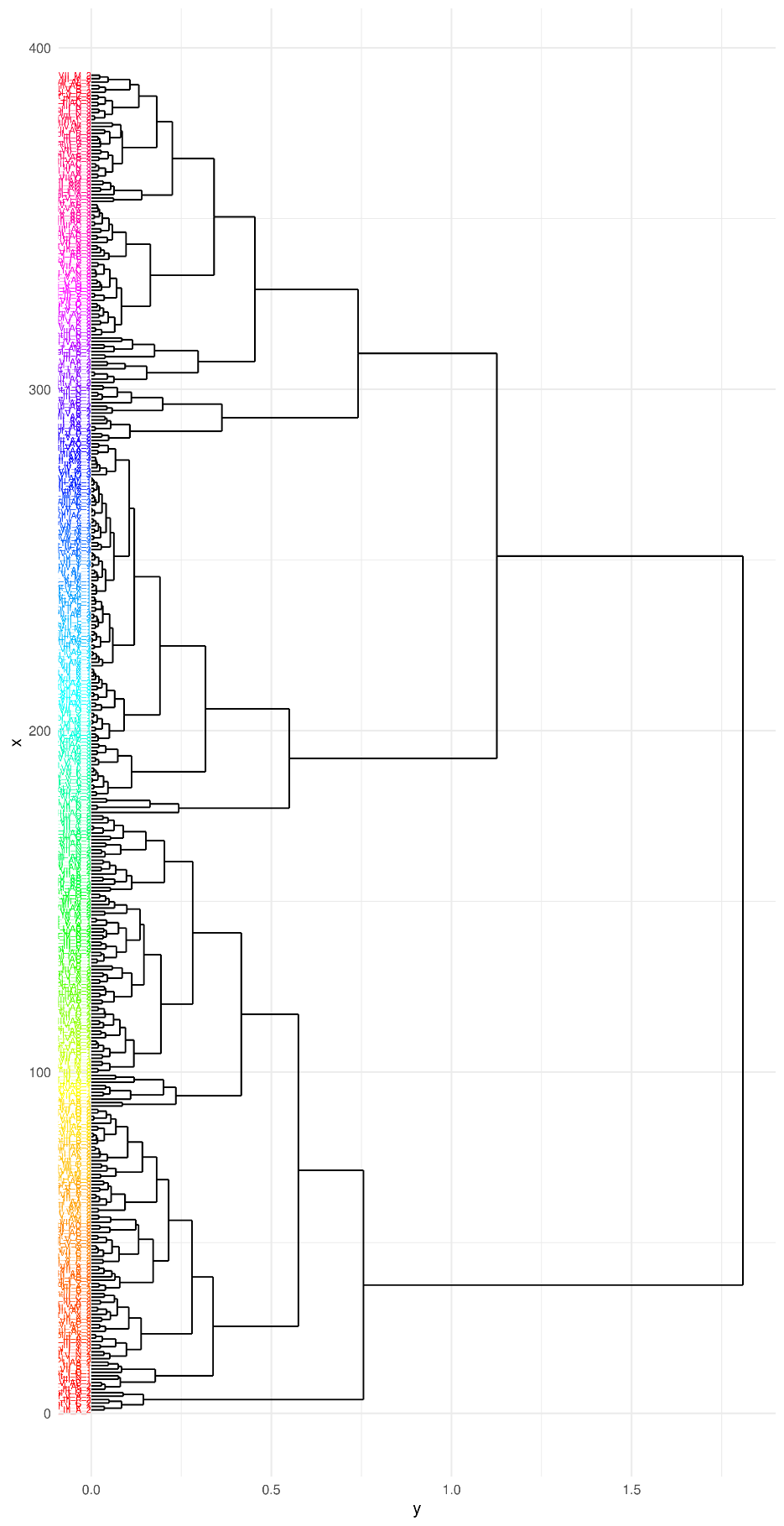


**Supplementary Figure S1.** Hierarchical Clustering Dendrogram of all individual samples (n = 396), showing how closely genotypes and replicates cluster within treatment, sampling time points, generation and endophyte presence.


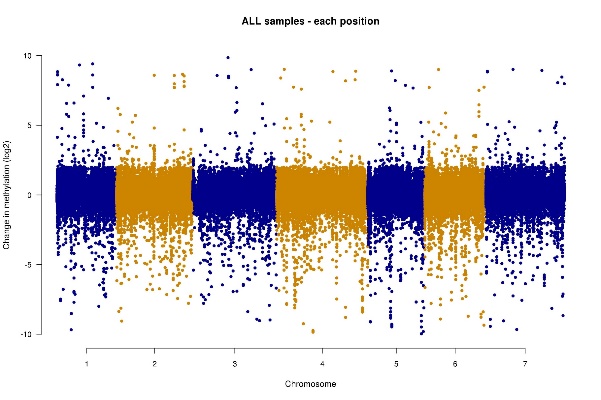


**Supplementary Figure S2.** The Manhattan plot displays the chromosomal distribution of differentially methylated regions (DMRs) among plants that are either endophyte-positive (E+) or endophyte-negative (E-). Each point on the plot represents an individual DMR, with its genomic location on the x-axis (e.g., chromosome and base pair position) and the log2 change in methylation in response to the presence or absence of endophytes on the y-axis.

# Supplementary Tables

| Generation | Samples | Samples after removal of low coverage | All unique sites * | Sites in one sample ** | Sites in 50% of the samples ** | Sites in 80% of the samples ** | Sites after removal of all NAs *** |
| --- | --- | --- | --- | --- | --- | --- | --- |
| G2 | 138 | 135 | 8,094,455 | 3,240,832 | 316,766 | 37,321 | 1,140 |
| G6 | 141 | 141 | 6,304,040 | 2,597,872 | 322,131 | 41,819 | 1,309 |
| G9 | 117 | 116 | 6,939,613 | 2,641,788 | 311,522 | 37,368 | 1,321 |
| ALL | **396** | **392** | **11,449,029** | **7,753,445** | **308,480** | **37,200** | **700** |

**Supplementary Table S1.** Occurrence of differential DNA methylated sites within plant generation.

* Number of methylated positions occurring exclusively in unique genomic sites across samples within each plant generation.

** Number of methylated positions occurring in genomic sites only in one sample, or in 50% and 80% of the samples, across all samples within each generation.

*** Number of methylated positions occurring in unique genomic sites within each generation after removing all missing values (NAs).

| Context | Genereation | Treatment | DMRs | Time Point | | | |
| --- | --- | --- | --- | --- | --- | --- | --- |
|  |  |  |  | I | II | III | IV |
| CG | G2 | Control | HYPERmethylated | 828 | 794 | 805 | 1125 |
| CG | G2 | Control | HYPOmethylated | 2102 | 2306 | 2679 | 1992 |
| CHG | G2 | Control | HYPERmethylated | 1065 | 939 | 1242 | 1830 |
| CHG | G2 | Control | HYPOmethylated | 3449 | 3875 | 4433 | 3215 |
| CHH | G2 | Control | HYPERmethylated | 1143 | 1123 | 1606 | 2139 |
| CHH | G2 | Control | HYPOmethylated | 4354 | 4755 | 5469 | 3781 |
| CG | G2 | Drought | HYPERmethylated | 910 | 899 | 837 | 920 |
| CG | G2 | Drought | HYPOmethylated | 2145 | 1957 | 2783 | 2601 |
| CHG | G2 | Drought | HYPERmethylated | 1300 | 1235 | 1268 | 1354 |
| CHG | G2 | Drought | HYPOmethylated | 3467 | 3045 | 4473 | 4295 |
| CHH | G2 | Drought | HYPERmethylated | 1456 | 1514 | 1541 | 1493 |
| CHH | G2 | Drought | HYPOmethylated | 4249 | 3870 | 5509 | 5199 |
| CG | G6 | Control | HYPERmethylated | 936 | 882 | 879 | 880 |
| CG | G6 | Control | HYPOmethylated | 2357 | 2332 | 3176 | 2442 |
| CHG | G6 | Control | HYPERmethylated | 1365 | 1382 | 1435 | 1454 |
| CHG | G6 | Control | HYPOmethylated | 3541 | 3327 | 5049 | 3946 |
| CHH | G6 | Control | HYPERmethylated | 1684 | 1769 | 1626 | 1709 |
| CHH | G6 | Control | HYPOmethylated | 4476 | 4136 | 6024 | 4922 |
| CG | G6 | Drought | HYPERmethylated | 850 | 888 | 866 | 1144 |
| CG | G6 | Drought | HYPOmethylated | 2468 | 2397 | 3138 | 2303 |
| CHG | G6 | Drought | HYPERmethylated | 1106 | 1275 | 1260 | 1727 |
| CHG | G6 | Drought | HYPOmethylated | 3936 | 3742 | 5060 | 3814 |
| CHH | G6 | Drought | HYPERmethylated | 1278 | 1396 | 1549 | 1913 |
| CHH | G6 | Drought | HYPOmethylated | 5099 | 4951 | 6214 | 4814 |
| CG | G9 | Control | HYPERmethylated | 1021 | 1060 | 928 | 1013 |
| CG | G9 | Control | HYPOmethylated | 2344 | 2412 | 3267 | 2340 |
| CHG | G9 | Control | HYPERmethylated | 1378 | 1597 | 1318 | 1551 |
| CHG | G9 | Control | HYPOmethylated | 3589 | 3570 | 5141 | 3673 |
| CHH | G9 | Control | HYPERmethylated | 1612 | 1900 | 1565 | 1805 |
| CHH | G9 | Control | HYPOmethylated | 4419 | 4550 | 6161 | 4744 |
| CG | G9 | Drought | HYPERmethylated | 1004 | 905 | 860 | 1066 |
| CG | G9 | Drought | HYPOmethylated | 2364 | 2319 | 3267 | 2035 |
| CHG | G9 | Drought | HYPERmethylated | 1441 | 1626 | 1331 | 1759 |
| CHG | G9 | Drought | HYPOmethylated | 3371 | 3267 | 4999 | 3120 |
| CHH | G9 | Drought | HYPERmethylated | 1457 | 1670 | 1559 | 2088 |
| CHH | G9 | Drought | HYPOmethylated | 4407 | 4429 | 5941 | 3899 |

**Supplementary Table S2.** Number of hypo- and hypermethylated DMR between endophyte-negative and endophyte-positive samples for each context, generation and treatment.

Sub-cluster 1

| Experimental groups | Effect | DFn | DFd | F | p | `p<.05` | ges |
| --- | --- | --- | --- | --- | --- | --- | --- |
| E-_G2 | DAS | 1 | 20984 | 0.526 | 0.47 |  | 0.0000 |
| E-_G2 | trt | 1 | 20984 | 19.4 | 0.00 | * | 0.0009 |
| E-_G6 | DAS | 1 | 20736 | 0.677 | 0.41 |  | 0.0000 |
| E-_G6 | trt | 1 | 20736 | 13.6 | 0.00 | * | 0.0007 |
| E-_G9 | DAS | 1 | 20449 | 0.138 | 0.71 |  | 0.0000 |
| E-_G9 | trt | 1 | 20449 | 9.51 | 0.00 | * | 0.0005 |
| E+_G2 | DAS | 1 | 18770 | 0.621 | 0.43 |  | 0.0000 |
| E+_G2 | trt | 1 | 18770 | 2.12 | 0.15 |  | 0.0001 |
| E+_G6 | DAS | 1 | 19848 | 7.04 | 0.01 | * | 0.0004 |
| E+_G6 | trt | 1 | 19848 | 1.11 | 0.29 |  | 0.0001 |
| E+_G9 | DAS | 1 | 19934 | 0.777 | 0.38 |  | 0.0000 |
| E+_G9 | trt | 1 | 19934 | 0.046 | 0.83 |  | 0.0000 |

Sub-cluster 2

| Experimental groups | Effect | DFn | DFd | F | p | `p<.05` | ges |
| --- | --- | --- | --- | --- | --- | --- | --- |
| E-_G2 | DAS | 1 | 40428 | 3302 | 0.00 | * | 0.0760 |
| E-_G2 | trt | 1 | 40428 | 144 | 0.00 | * | 0.0040 |
| E-_G6 | DAS | 1 | 40223 | 3666 | 0.00 | * | 0.0840 |
| E-_G6 | trt | 1 | 40223 | 77.3 | 0.00 | * | 0.0020 |
| E-_G9 | DAS | 1 | 39559 | 3555 | 0.00 | * | 0.0820 |
| E-_G9 | trt | 1 | 39559 | 11.8 | 0.00 | * | 0.0003 |
| E+_G2 | DAS | 1 | 35816 | 1564 | 0.00 | * | 0.0420 |
| E+_G2 | trt | 1 | 35816 | 691 | 0.00 | * | 0.0190 |
| E+_G6 | DAS | 1 | 38207 | 3887 | 0.00 | * | 0.0920 |
| E+_G6 | trt | 1 | 38207 | 9.67 | 0.00 | * | 0.0003 |
| E+_G9 | DAS | 1 | 38264 | 5044 | 0.00 | * | 0.1160 |
| E+_G9 | trt | 1 | 38264 | 24.8 | 0.00 | * | 0.0006 |

Sub-cluster 3

| Experimental groups | Effect | DFn | DFd | F | p | `p<.05` | ges |
| --- | --- | --- | --- | --- | --- | --- | --- |
| E-_G2 | DAS | 1 | 36211 | 2328 | 0.00 | * | 0.0600 |
| E-_G2 | trt | 1 | 36211 | 47.6 | 0.00 | * | 0.0010 |
| E-_G6 | DAS | 1 | 35504 | 2618 | 0.00 | * | 0.0690 |
| E-_G6 | trt | 1 | 35504 | 0.44 | 0.51 |  | 0.0000 |
| E-_G9 | DAS | 1 | 34842 | 2525 | 0.00 | * | 0.0680 |
| E-_G9 | trt | 1 | 34842 | 53.6 | 0.00 | * | 0.0020 |
| E+_G2 | DAS | 1 | 33688 | 1325 | 0.00 | * | 0.0380 |
| E+_G2 | trt | 1 | 33688 | 914 | 0.00 | * | 0.0260 |
| E+_G6 | DAS | 1 | 35240 | 2261 | 0.00 | * | 0.0600 |
| E+_G6 | trt | 1 | 35240 | 1.03 | 0.31 |  | 0.0000 |
| E+_G9 | DAS | 1 | 35535 | 4045 | 0.00 | * | 0.1020 |
| E+_G9 | trt | 1 | 35535 | 64 | 0.00 | * | 0.0020 |

Sub-cluster 4

| Experimental groups | Effect | DFn | DFd | F | p | `p<.05` | ges |
| --- | --- | --- | --- | --- | --- | --- | --- |
| E-_G2 | DAS | 1 | 3308 | 466 | 0.00 | * | 0.1230 |
| E-_G2 | trt | 1 | 3308 | 0.495 | 0.48 |  | 0.0002 |
| E-_G6 | DAS | 1 | 3272 | 724 | 0.00 | * | 0.1810 |
| E-_G6 | trt | 1 | 3272 | 2.99 | 0.08 |  | 0.0009 |
| E-_G9 | DAS | 1 | 3205 | 638 | 0.00 | * | 0.1660 |
| E-_G9 | trt | 1 | 3205 | 51.3 | 0.00 | * | 0.0160 |
| E+_G2 | DAS | 1 | 2842 | 329 | 0.00 | * | 0.1040 |
| E+_G2 | trt | 1 | 2842 | 202 | 0.00 | * | 0.0660 |
| E+_G6 | DAS | 1 | 3014 | 815 | 0.00 | * | 0.2130 |
| E+_G6 | trt | 1 | 3014 | 0.016 | 0.90 |  | 0.0000 |
| E+_G9 | DAS | 1 | 3102 | 1268 | 0.00 | * | 0.2900 |
| E+_G9 | trt | 1 | 3102 | 5.88 | 0.02 | * | 0.0020 |

**Supplementary Table S3.** Main effect analyses for treatment at different levels of experimental groups for each sub-cluster. DFn: degrees of freedom for the numerator; DFd: degrees of freedom for the denominator; F: F-statitics; p: p-value; p<.05 denote significance; ges: generalized eta squared, an effect size measurement.
